# Supplementary material for: Foraging dynamics are associated with social status and context in mouse social hierarchies
Source: PeerJ. 2018 Sep 19;6:e5617. doi: 10.7717/peerj.5617 (PMC6151111; doi:10.7717/peerj.5617)

**Supplementary Figures**

**Supplemental Figure S1.** Vivarium for social group housing. This photo was taken by James Curley.

**
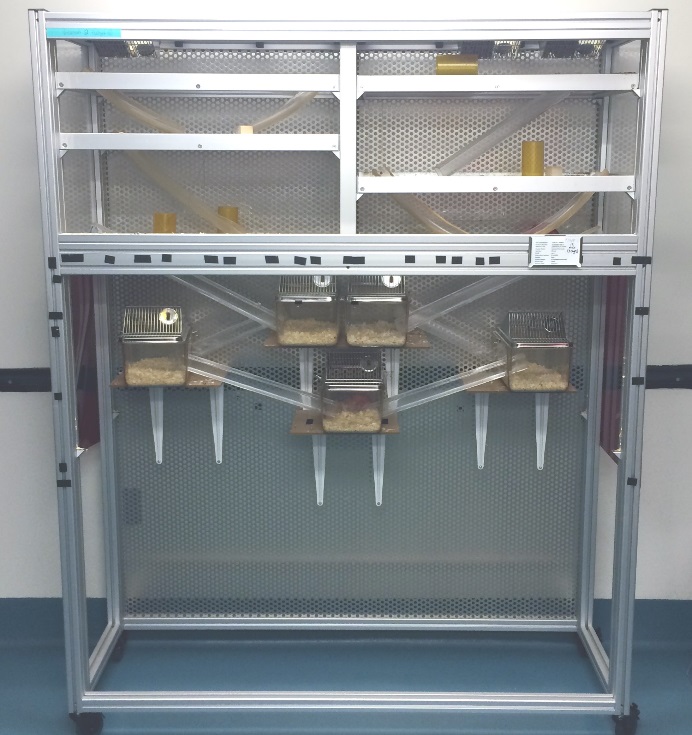
**

**Supplemental Figure S2**. Temporal dynamics of individual Glicko ratings throughout entire group housing period. Yellow shades indicate the aggressive interaction events that occurred on the day of video recording. Videos recording foraging behavior were sampled on Day 1 of group housing from 5 cohorts (Day 1) and on between Day 6 and 22 (Stable).


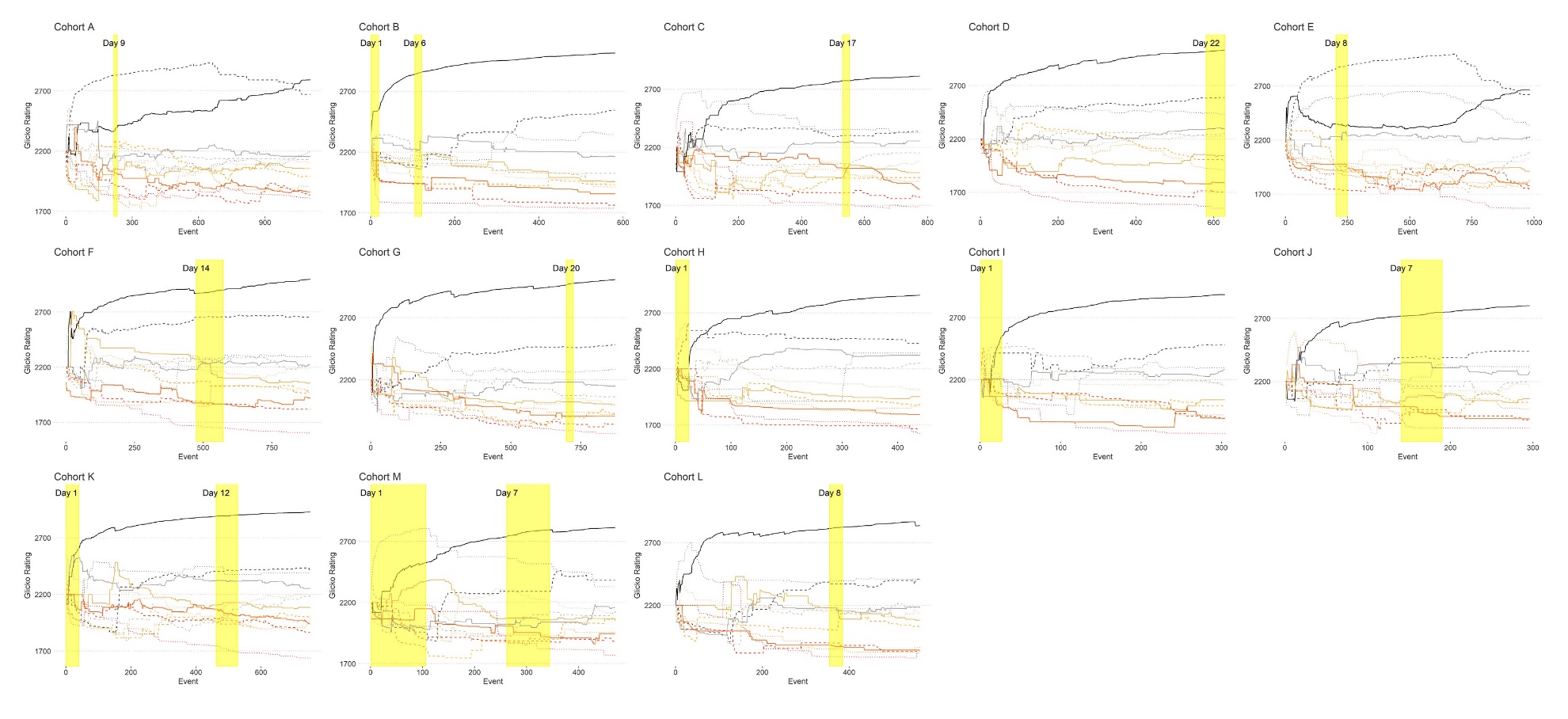


**Supplemental Figure S3.** The differences in eating and drinking frequency among social status groups on the first day of group housing (Day 1) and after social hierarchies were established (Stable). Individuals in the alpha group are those with Glicko rank 1 on the day of video recording. Individuals with Glicko rating over 2200 (initial starting point, see method for details) belong to the subdominant social group and those with Glicko rating less than 2200 are in the subordinate group.


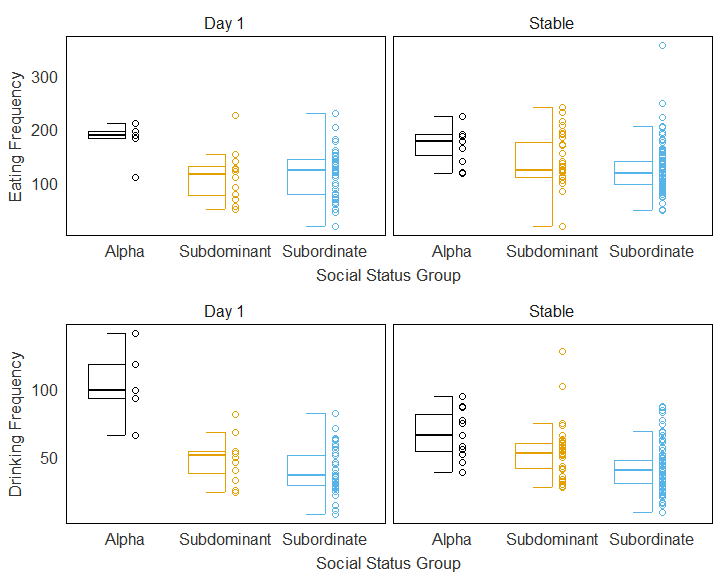


**Supplemental Figure S4.** Duration of eating and drinking by social rank on Day 1 and after the hierarchies have stabilized.


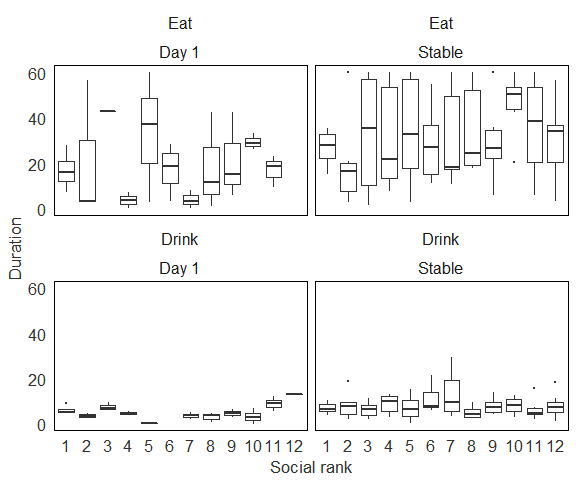


**Supplemental Figure S5.** The effect of social status on (a) the percentage of feeding and drinking bouts during the light phase across days (b) the relative density distribution of eating and drinking activity across days (the red hashed line indicates the time of the change from dark to light phase).

(a)


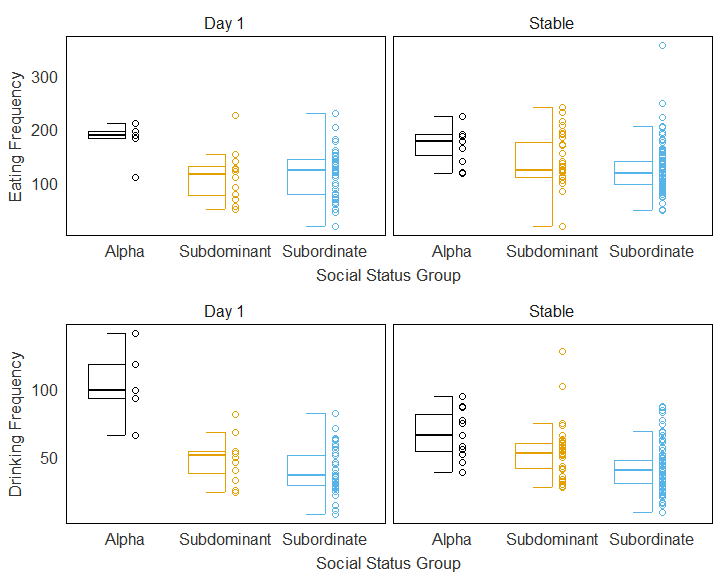


(b)


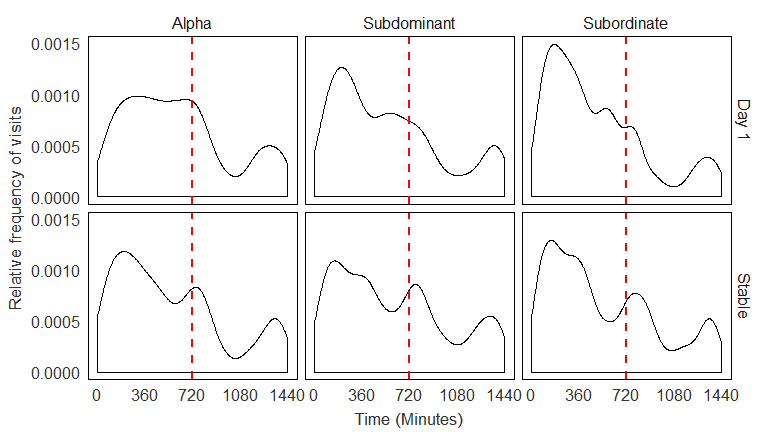


**Supplemental Figure S6.** Heat maps of the frequency of visits to food hoppers by location of each cohort by rank


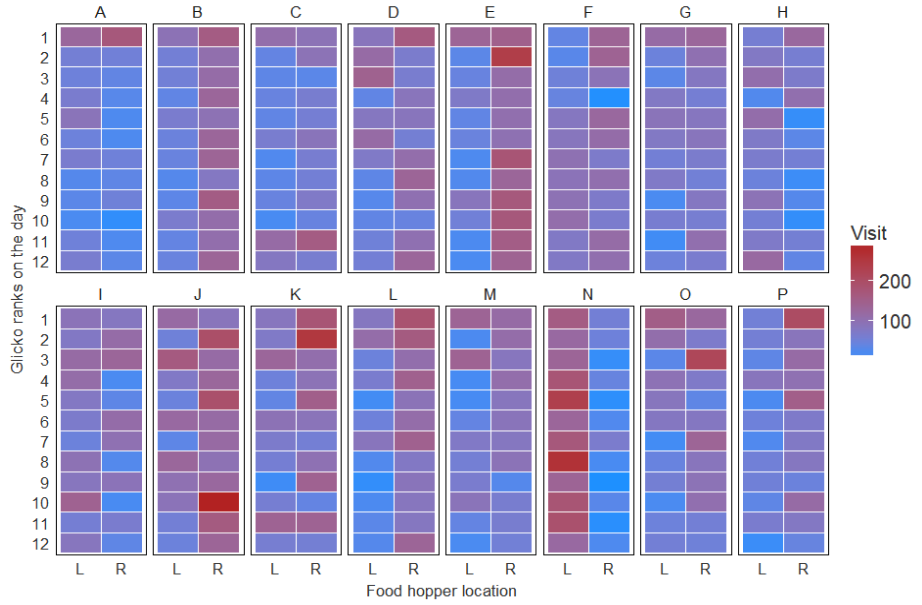

Supplement: Supplemental Information 1 [file peerj-06-5617-s001.docx]
