# Supplementary material for: Foraging dynamics are associated with social status and context in mouse social hierarchies
Source: PeerJ. 2018 Sep 19;6:e5617. doi: 10.7717/peerj.5617 (PMC6151111; doi:10.7717/peerj.5617)
Supplement: Supplemental Information 2 [file peerj-06-5617-s002.docx]

**Supplemental Tables**

**Supplemental Table S1. Mouse social behavior ethogram**

| Priority | Behavior | Description |
| --- | --- | --- |
| 1 | Fighting | Individual lunges at and/or bites the other individual |
|  |  |  |
| 2 | Chasing | Individual follows the target individual rapidly and aggressively while the other individual attempts to flee |
|  |  |  |
| 3 | Mounting | Individual mounts another individual from behind with the recipient attempting to flee or otherwise being pinned to the floor |
|  |  |  |
| 4 | Subordinate posture | Individual responds to the approach from another individual by remaining motionless and/or exposing their nape |
|  |  |  |
| 5 | Induced-flee | Individual flees without any aggression shown by another individual |

**Supplemental Table S2. Glicko Ratings of individuals (a) on Day1 and (b) after hierarchies were established. Individuals with no ratings did not engage in any agonistic interaction on Day1.**

**(a)**

| **Glicko Rank** | **Group A** | **Group B** | **Group C** | **Group D** | **Group E** |
| --- | --- | --- | --- | --- | --- |
| **1** | **2546** | **2543** | **2548** | **2608** | **2807** |
| **2** | **2342** | **2441** | **2458** | **2524** | **2527** |
| **3** | **2315** | **2220** | **2415** | **2334** | **2379** |
| **4** | **2179** | **2130** | **2211** | **2193** | **2180** |
| **5** | **2130** | **2118** | **2208** | **2130** | **2149** |
| **6** | **2124** | **2107** | **2144** | **2068** | **2006** |
| **7** | **2106** | **2095** | **2089** | **2064** | **2003** |
| **8** | **1996** | **2091** | **2087** | **2037** | **1982** |
| **9** | **1983** | **2040** | **2065** | **1972** | **1981** |
| **10** | **1790** | **1915** | **2006** | **1965** | **1956** |
| **11** | **NA** | **NA** | **1998** | **1928** | **1895** |
| **12** | **NA** | **NA** | **NA** | **NA** | **1839** |

**(b)**

| **Glicko rank** | **Group**  **F** | **Group**  **G** | **Group H** | **Group I** | **Group J** | **Group K** | **Group L** | **Group M** | **Group N** | **Group O** | **Group P** |
| --- | --- | --- | --- | --- | --- | --- | --- | --- | --- | --- | --- |
| **1** | **2841** | **2861** | **2796** | **3054** | **2893** | **2919** | **3003** | **2753** | **2920** | **2834** | **2807** |
| **2** | **2395** | **2282** | **2338** | **2610** | **2595** | **2679** | **2474** | **2389** | **2422** | **2396** | **2510** |
| **3** | **2276** | **2173** | **2289** | **2422** | **2344** | **2294** | **2263** | **2339** | **2368** | **2377** | **2293** |
| **4** | **2229** | **2158** | **2250** | **2310** | **2240** | **2280** | **2188** | **2307** | **2318** | **2176** | **2203** |
| **5** | **2159** | **2139** | **2073** | **2252** | **1981** | **2236** | **2062** | **2162** | **2136** | **2151** | **2130** |
| **6** | **2056** | **2129** | **2060** | **2232** | **1952** | **2142** | **2058** | **2101** | **2125** | **2136** | **2052** |
| **7** | **2051** | **2081** | **2003** | **2050** | **1940** | **2067** | **2021** | **2078** | **2080** | **2133** | **2043** |
| **8** | **2043** | **2061** | **1956** | **1975** | **1936** | **2036** | **1934** | **2070** | **2001** | **2083** | **1931** |
| **9** | **2018** | **1994** | **1954** | **1897** | **1923** | **2018** | **1901** | **1977** | **1964** | **1880** | **1916** |
| **10** | **1906** | **1943** | **1943** | **1788** | **1905** | **1864** | **1897** | **1954** | **1945** | **1867** | **1903** |
| **11** | **1829** | **1938** | **1806** | **1701** | **1873** | **1860** | **1845** | **1921** | **1937** | **1846** | **1882** |
| **12** | **1801** | **1782** | **1698** | **1541** | **1732** | **1641** | **1782** | **1829** | **1676** | **1785** | **1818** |
